# Sustainable Soil–Cement Composites with Rice Husk Ash and Silica Fume: A Review of Performance and Environmental Benefits

**DOI:** 10.3390/ma18122880

**Published:** 2025-06-18

**Authors:** Xiaosan Yin, Md Mashiur Rahman, Yuzhou Sun, Yi Zhao, Jian Wang

**Affiliations:** 1 School of Intelligent Construction and Civil Engineering, Zhongyuan University of Technology, Zhengzhou 451191, China; mramoshi255@outlook.com (M.M.R.); 6357@zut.edu.cn (Y.Z.); 2024109018@zut.edu.cn (J.W.); 2 Henan Mechanics and Structures Engineering Research Centre, Zhengzhou 451191, China; 3 School of Civil and Transportation Engineering, Henan University of Urban Construction, Pingdingshan 467036, China; sunyz@zut.edu.cn

**Keywords:** rice husk ash, silica fume, sustainable construction, supplementary cementitious materials, circular economy

## Abstract

The construction industry urgently requires sustainable alternatives to conventional cement to mitigate its environmental footprint, which includes 8% of global CO_2_ emissions. This review critically examines the potential of rice husk ash (RHA) and silica fume (SF)—industrial and agricultural byproducts—as high-performance supplementary cementitious materials (SCMs) in soil–cement composites. Their pozzolanic reactivity, microstructural enhancement mechanisms, and durability improvements (e.g., compressive strength gains of up to 31.7% for RHA and 250% for SF) are analyzed. This study highlights the synergistic effects of RHA/SF blends in refining pore structure, reducing permeability, and enhancing resistance to chemical attacks. Additionally, this paper quantifies the environmental benefits, including CO_2_ emission reduction (up to 25% per ton of cement replaced) and resource recovery from agricultural/industrial waste streams. Challenges such as material variability, optimal dosage (10–15% RHA, 5–8% SF), and regulatory barriers are discussed, alongside future directions for scalable adoption. This work aligns with SDGs 9, 11, and 12, offering actionable insights for sustainable material design.

## 1. Introduction

The construction industry is one of the largest contributors to global environmental degradation, accounting for significant greenhouse gas emissions, resource depletion, and waste generation. Cement production, a cornerstone of modern construction, is responsible for approximately 8% of global CO_2_ emissions, making it a critical target for sustainable innovation [1]. As urbanization and infrastructure development continue to accelerate worldwide, the demand for cement and other construction materials is expected to rise exponentially. This growing demand exacerbates environmental challenges, including the depletion of natural resources, increased energy consumption, and construction and demolition waste generation. Therefore, the urgent need for sustainable alternatives to traditional construction materials has never been more pressing. Supplementary cementitious materials (SCMs) have emerged as viable solutions to reduce the environmental footprint of the construction industry. SCMs, when used in combination with cement, enhance the properties of concrete and other cement-based composites while reducing reliance on traditional Portland cement [2]. Among the most promising SCMs are rice husk ash (RHA) and silica fume (SF), both of which are byproducts of industrial and agricultural processes. RHA, derived from the combustion of rice husks, typically results from rice milling, while SF is a byproduct of silicon and ferrosilicon alloy production [3]. These materials not only provide sustainable alternatives to cement but also address waste management problems by repurposing agricultural and industrial byproducts. RHA and SF exhibit pozzolanic properties, meaning that they can react with calcium hydroxide in the presence of moisture to form additional calcium silicate hydrate (C-S-H) compounds [4]:

SiO_2_ (RHA/SF) + Ca(OH)_2_ → C-S-H

This reaction enhances the mechanical and durability properties of cement-based composites, making them stronger, more durable, and more resistant to environmental degradation. For instance, RHA can improve compressive strength by up to 31.7%, while SF can enhance it by 250% compared to traditional cement mixes [5]. Additionally, these materials improve resistance to chemical attacks, freeze–thaw cycles, and sulfate exposure [6], making them suitable for harsh environments such as coastal areas and regions with extreme weather conditions [7]. Studies have highlighted that the incorporation of RHA, especially when combined with other waste materials, such as fly ash, can yield exceptional mechanical strength and durability due to the synergistic effects of mixed recycled materials [8].

Beyond their mechanical and durability benefits, RHA and SF offer significant environmental advantages. The use of these materials promotes resource efficiency by repurposing waste that would otherwise contribute to landfill volume and environmental pollution. For example, the global production of rice husks exceeds 120 million tons annually, much of which is discarded or burned in open fields, significantly contributing to air pollution [9]. The primary hydration reaction is as follows:

C_3_S + H_2_O → C-S-H + Ca(OH)_2_

**Figure 1:**
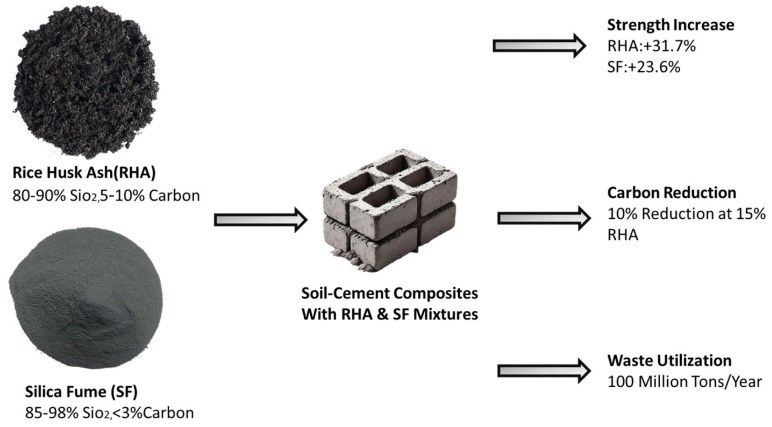
Sustainable soil–cement composites using RHA and SF.

By incorporating RHA into construction materials, this waste can be transformed into a valuable resource, aligning with the principles of a circular economy. Similarly, SF utilizes byproducts from silicon production, reducing the environmental impact of industrial processes. The adoption of RHA and SF also contributes to carbon emission reduction. Cement manufacturing is an energy-intensive process that requires high temperatures (1400–1500 °C) and emits approximately 0.9 tons of CO_2_ per ton of cement produced [10]. By replacing a portion of cement with RHA and SF, the construction industry can significantly lower its carbon footprint. For instance, a 15% replacement of cement with RHA can reduce CO_2_ emissions by up to 10% [11]. This reduction is particularly significant in developing countries, where cement production is a major contributor to greenhouse gas emissions [12]. Despite their numerous benefits, the widespread adoption of RHA and SF faces several challenges. Material variability and quality control are significant concerns, as the chemical composition and pozzolanic activity of RHA and SF can vary based on factors such as combustion temperature and production processes [13]. Standardized testing methods and quality control protocols are essential to ensure consistent performance. Additionally, determining the optimal dosage of RHA and SF is critical for maximizing performance. While RHA can replace up to 25% of cement, the optimal replacement level is typically around 15%. Similarly, SF is most effective at dosages of 2–6% [14]. Figure 1 shows the need of further research to develop guidelines for different soil types and environmental conditions [15].

## 2. Characteristics of Rice Husk Ash and Silica Fume

### 2.1. Chemical Composition

The chemical composition of rice husk ash (RHA) and silica fume (SF) fundamentally determines their effectiveness as supplementary cementitious materials (SCMs). Both materials are classified as highly reactive pozzolans due to their high amorphous silica (SiO_2_) content, typically exceeding 85% by mass [16]. This non-crystalline silica exists in a metastable state that readily participates in pozzolanic reactions with calcium hydroxide during cement hydration. However, their chemical profiles differ significantly due to distinct origins and production processes. RHA composition varies based on rice species, combustion conditions (500–800 °C), and processing methods, typically containing 85–95% SiO_2_, along with minor amounts of Al_2_O_3_ (0.5–3%), Fe_2_O_3_ (0.5–3%), and alkaline oxides (1–5%) [17]. In contrast, SF exhibits a more consistent composition (90–98% SiO_2_) due to the controlled industrial production of silicon alloys, with minimal impurities [18] as shown in Table 1.

### 2.2. Mechanical Properties

The incorporation of rice husk ash (RHA) and silica fume (SF) as supplementary cementitious materials significantly enhances the mechanical performance of cement-based composites. Extensive research has demonstrated their ability to improve compressive strength, tensile capacity, and deformation characteristics through distinct yet complementary mechanisms [19]. These improvements are particularly valuable for structural applications where enhanced load-bearing capacity and durability are required.

Compressive strength’s development follows different patterns for RHA- and SF-modified systems. RHA typically shows a gradual strength increase, with optimal performance achieved at 10–25% cement replacement, resulting in a 20–31.7% strength improvement at 28 days [20]. This sustained strength gain continues beyond 90 days due to RHA’s prolonged pozzolanic activity. In contrast, SF-modified systems exhibit rapid early strength development, with 5–10% replacement yielding a 23.6–35% strength enhancement, primarily attributed to its ultra-fine particles filling nano-scale voids in the cement matrix [21]. The fibrous structure of RHA contributes to crack bridging, while SF’s nano-filler effect produces a denser microstructure with reduced porosity.

The tensile and flexural performance of cementitious composites also benefits substantially from the incorporation of RHA and SF. RHA’s natural fiber-like morphology enhances energy absorption, with 15% replacement increasing flexural strength by 18–25% while reducing brittle failure tendencies [22]. SF improves interfacial transition zone bonding between cement paste and aggregates, leading to 20–28% higher splitting tensile strength at just a 5% addition [23]. Both materials effectively minimize microcrack propagation, albeit through different mechanisms—RHA through its particle morphology, and SF through pore refinement. Further studies indicate that unconventional fibers, when combined with these SCMs, could yield additional enhancements in tensile performance under dynamic loading conditions [24]. The elastic modulus and deformation characteristics show interesting variations between RHA- and SF-modified systems. RHA typically increases the elastic modulus by 10–15% while maintaining better strain capacity than plain cement, resulting in improved toughness indices [25]. SF systems demonstrate more pronounced stiffness enhancement (15–25%) and develop more linear stress–strain behavior, along with superior creep resistance [26].

Moreover, the incorporation of RHA and SF has been shown to enhance the resilience of concrete during extreme stress conditions, making them suitable for infrastructure projects in seismic areas or regions affected by heavy loading [27]. Recent studies show that RHA not only contributes to improved compressive strength but also reinforces the material’s ductility, which is crucial for structures that are prone to dynamic loading [28].

Recent studies have revealed promising synergistic effects when RHA and SF are used in combination. Optimal blends of 10% RHA with 5% SF have demonstrated 35–45% compressive strength improvements over control mixes [29]. The sequential reactivity of these materials—with SF providing early strength gain and RHA contributing to long-term development—creates a continuous strengthening mechanism. This combination approach has shown particular promise in high-performance applications where both immediate and sustained mechanical performance are critical. As shown in Table 2, RHA demonstrated a 31.7% strength increase at 10% dosage, while SF achieved a dramatic 250% enhancement at a 6% loading. Nano-silica exhibited the most pronounced effect, reaching a 323% strength gain at just a 3% dosage [30]. These results suggest that material selection should consider both performance peaks and economic feasibility.

**Figure 2:**
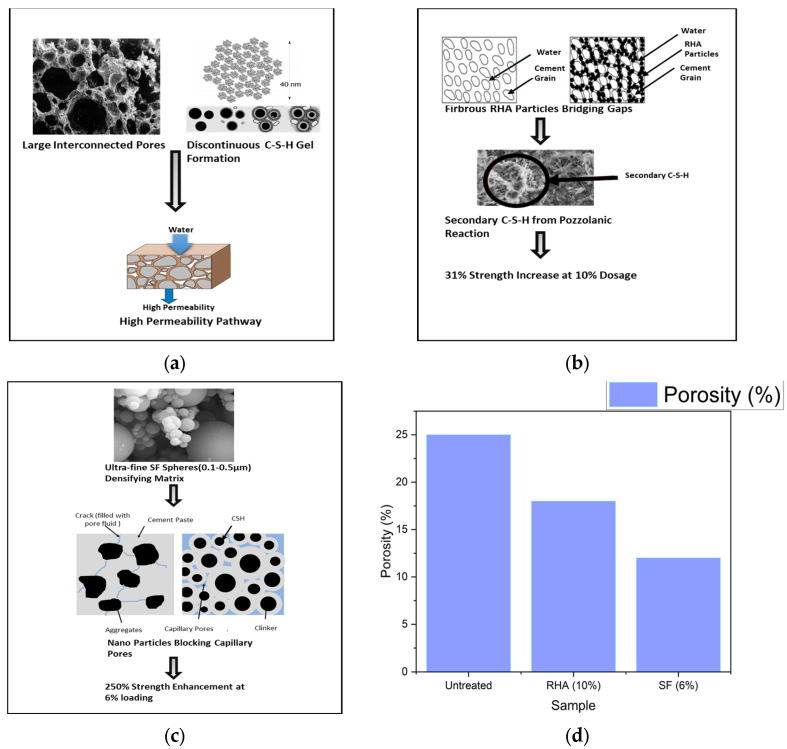
Mechanisms of porosity and permeability reduction in RHA/SF-modified soil–cement composites: (**a**) Untreated soil–cement. (**b**) RHA-treated composites. (**c**) SF-treated composites. (**d**) Porosity reduction [5,6,7,8,9].

The untreated soil–cement exhibits higher porosity and permeability, which can lead to lower strength and durability. In contrast, the RHA-treated composites demonstrate a significant reduction in porosity due to the pozzolanic reaction of RHA with calcium hydroxide, forming additional calcium silicate hydrate (C-S-H) gel (Figure 2). This reaction enhances the microstructure, leading to improved strength and durability [5,6]. Similarly, SF-treated composites show a marked decrease in porosity, attributed to the ultra-fine particles of SF that fill capillary pores and densify the microstructure. The overall porosity reduction highlights the effectiveness of both RHA and SF in enhancing the mechanical properties and durability of soil–cement composites [7,8].

### 2.3. Durability Enhancement

The long-term performance of cementitious materials in aggressive environments is significantly enhanced through the incorporation of rice husk ash (RHA) and silica fume (SF), which improve durability via synergistic mechanisms. Research demonstrates that SF’s ultra-fine particles (0.1–0.5 μm) effectively refine capillary pores, reducing water permeability by 40–50% compared to conventional concrete, while RHA’s pozzolanic reaction products gradually clog interconnected pores, decreasing water absorption by 30–35% after 90 days [31]. Field studies confirm that SF-modified concrete exhibits chloride diffusion coefficients 50% lower than those of reference mixes after five years in marine environments [32]. In terms of chemical resistance, RHA–SF blends mitigate sulfate attack by reducing calcium hydroxide content and limiting sulfate penetration, resulting in 60–70% lower expansion [33]. Acid resistance is also improved, with modified C-S-H structures demonstrating lower solubility in acidic conditions (pH 2–4), extending their service life in wastewater plants by threefold [34]. Additionally, recent studies have indicated that the combination of RHA and SF can effectively enhance the carbonation resistance of concrete. Table 3 shows that property is particularly important in minimizing potential structural degradation over time due to CO_2_ exposure [35]. The blending of these materials has also proven effective in landfill applications, where their leachate control properties can mitigate the environmental impacts of heavy metal contaminants [36].

Alkali–silica reactions are suppressed through reduced alkali availability and a denser microstructure, particularly at replacement levels exceeding 15% RHA and 7% SF [25]. Environmental durability is enhanced; freeze–thaw resistance improves by 3–4 times due to the refined pore structure [21], while the carbonation depth is reduced by 30–40% at optimal replacement levels [22]. Moreover, chloride-induced corrosion is delayed by 5–8 times in marine settings, with synergistic effects observed in combined RHA–SF systems [19]. These findings underscore the effectiveness of RHA and SF in enhancing the durability of cementitious materials in harsh conditions, supporting their use in sustainable construction. Additionally, recent studies have indicated that the combination of RHA and SF can effectively enhance the carbonation resistance of concrete. This property is particularly important in minimizing potential structural degradation over time due to CO_2_ exposure [31]. The blending of these materials has also proven effective in landfill applications, where their leachate control properties can mitigate the environmental impacts of heavy metal contaminants [37].

## 3. Environmental Impact

The environmental benefits of incorporating rice husk ash (RHA) and silica fume (SF) in construction materials extend far beyond waste reduction, offering comprehensive solutions to some of the most pressing sustainability challenges in the construction sector. This section provides a detailed examination of three critical environmental dimensions: (1) The transformation of agricultural and industrial waste into valuable resources through circular economy principles, (2) the significant reduction in carbon emissions compared to conventional cement production, and (3) the improvement of soil properties and erosion control in geotechnical applications. The analysis draws upon recent life-cycle assessment studies and field implementation data to present a holistic view of the environmental advantages offered by these supplementary cementitious materials.

### 3.1. Waste Utilization and Circular Economy

The utilization of RHA and SF represents a paradigm shift in construction material sourcing, transforming linear waste streams into circular resource flows. Global rice production generates approximately 120 million tons of husks annually, with traditional disposal methods like open burning contributing significantly to air pollution—emitting an estimated 1.5 kg of CO_2_ per kilogram of husk burned, along with substantial particulate matter (PM_2.5_) [38]. The conversion of these husks into RHA for construction applications prevents 8–12 tons of CO_2_ equivalent emissions per ton of husk, compared to conventional disposal methods. Similarly, the ferrosilicon industry produces about 2 million tons of SF annually as a byproduct, which, when utilized in concrete, can replace 5–10% of cement content without requiring additional processing [37]. This direct substitution approach yields energy savings of 15–18 gigajoules per ton compared to virgin silica production, while simultaneously addressing the challenge of industrial byproduct management [39].

**Figure 3:**
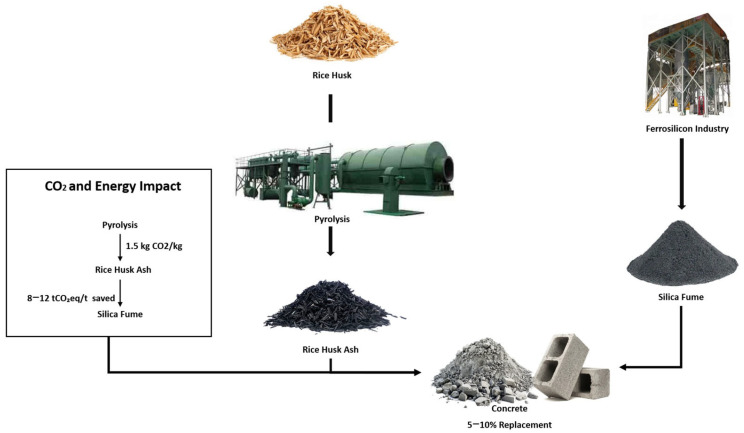
Material flows in RHA/SF utilization. Outer ring shows mass balances (120 M ton husks → 102 M t RHA at 85% yield); inner ring displays CO_2_ savings (8–12 tCO_2_eq/t vs. burning) [37,38,39].

**Figure 4:**
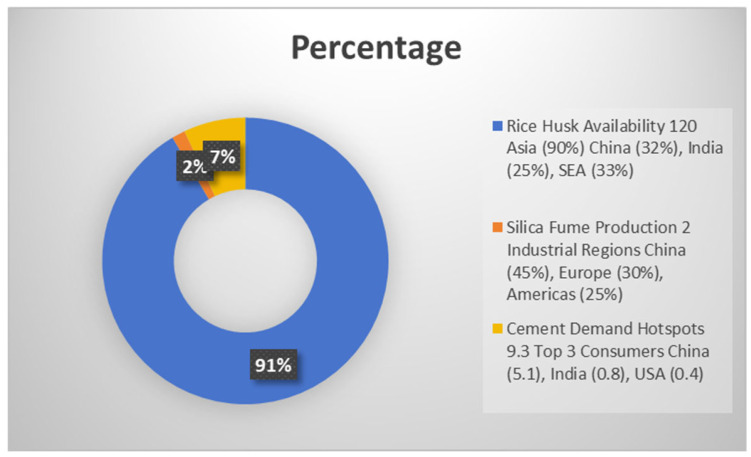
Global material flow matrix—Geographic distribution of rice husk ash potential, silica fume production, and cement demand hotspots [1,2,3].

Figure 3 shows the circular economy model implemented through the utilization of RHA and SF demonstrates remarkable efficiency, with material yield rates of 85% for RHA production from rice husks and 92% for SF incorporation in concrete mixtures [1]. These processes not only reduce landfill burdens but also create new economic value chains, particularly in agricultural regions where rice husks are abundantly available. Field studies in Southeast Asia have shown that localized RHA production systems can reduce transportation-related emissions by up to 40% compared to imported cementitious materials, while also creating rural employment opportunities in material processing and quality control [2]. The environmental benefits are further amplified when considering the entire life cycle; RHA- and SF-modified constructions demonstrate extended service life and reduced maintenance requirements due to their enhanced durability characteristics. Utilizing these materials also aligns with international environmental agreements aimed at reducing waste and promoting sustainable practices within the industry [3] (Figure 4).

### 3.2. Carbon Emissions Reduction

**Figure 5:**
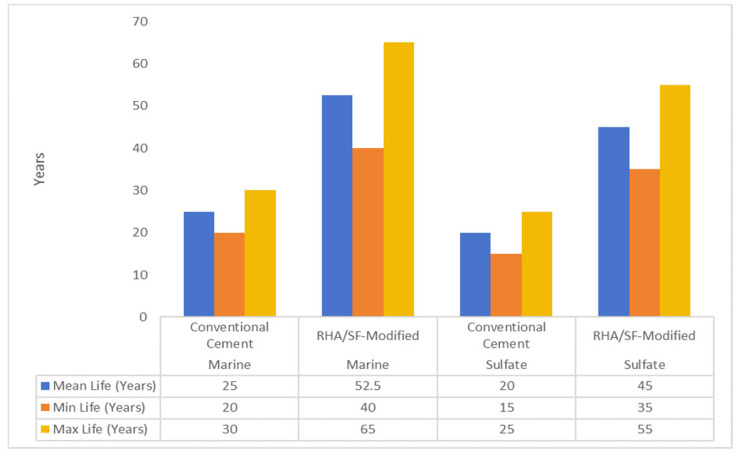
Service life extension projections for marine and sulfate-rich environments [6].

The carbon reduction potential of RHA and SF stems from multiple synergistic mechanisms that address both process emissions and material efficiency. Cement production remains one of the most carbon-intensive industrial processes, emitting approximately 0.89 kg of CO_2_ per kilogram of Portland cement produced, with clinker formation alone accounting for about 60% of these emissions [4]. RHA and SF offer distinct but complementary pathways for decarbonization. RHA production through controlled combustion emits only 0.18 kg of CO_2_ per kilogram, representing an 80% reduction compared to cement, while SF utilization is even more favorable, as it repurposes an existing industrial byproduct with minimal additional processing emissions [5]. At typical replacement levels of 15–25% for RHA and 5–10% for SF, blended cement systems can achieve 20–35% reductions in embodied carbon per cubic meter of concrete. The microstructural modifications induced by these materials contribute to additional carbon savings through enhanced durability—laboratory tests and field observations indicate that RHA/SF-modified concrete exhibits 30–50% lower chloride permeability and 40–60% greater resistance to sulfate attack, significantly extending its service life in aggressive environments [6]. As illustrated in Figure 5 accelerated testing data project service life extensions of 40–65 years (vs. 20–30 years for conventional cement) in marine environments and 35–55 years (vs. 15–25 years) in sulfate-rich conditions, with error ranges reflecting experimental variability (ASTM C1202/C1012 [40,41]).

This durability enhancement translates to reduced material consumption over the life cycle of structures. Life-cycle assessment studies demonstrate 15–25% lower carbon emissions per year of service compared to conventional concrete [7]. Furthermore, the lower curing temperatures required for RHA/SF-modified mixes (typically 20–30 °C less than standard concrete) contribute to additional energy savings during construction [8]. In developing countries, where the demand for cement is growing rapidly, the widespread adoption of these materials could reduce projected sector emissions by 8–12% by 2030 while meeting infrastructure development needs [9]. Furthermore, the integration of rice husk ash and silica fume can lead to a circular economy model that reduces the carbon footprint of construction through minimizing waste and improving resource recycling [10]. Table 4 shows recent advancements in carbon accounting methods, particularly in regions with stringent environmental policies, may incentivize further incorporation of RHA and SF by demonstrating tangible reductions in carbon emissions tied to their use [11].

### 3.3. Improved Soil Properties and Erosion Control

**Figure 6:**
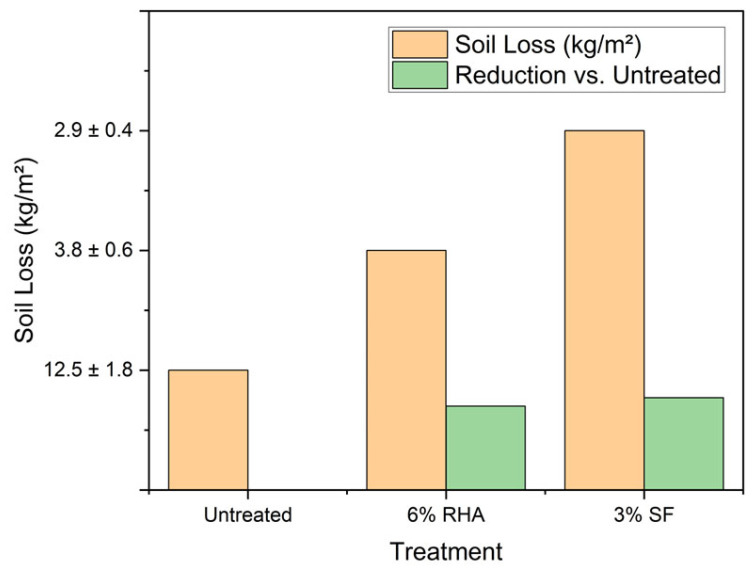
Comparative performance of RHA- and SF-stabilized slopes under an extreme rainfall simulation (100-year storm event) [20,21].

Beyond their applications in conventional concrete, RHA and SF demonstrate remarkable potential for sustainable geotechnical applications, particularly in soil stabilization and erosion control. The pozzolanic reactions of these materials with natural soils create stable, cementitious matrices that improve both mechanical properties and environmental resilience [12]. Laboratory tests show that the addition of 6–8% RHA to clayey soils can reduce the plasticity index by 30–40% while increasing unconfined compressive strength by 200–300% after 28 days of curing [13]. These modifications make treated soils more suitable for construction while reducing the need for expensive imported aggregates. RHA’s lightweight nature, coupled with its ability to improve mechanical attributes, positions it as an effective stabilizer for a variety of soil types. Furthermore, the combination of RHA and other organic additives has been found to increase the moisture retention ability of treated soils, which can be particularly beneficial in arid regions [14]. In erosion-prone areas, field trials have demonstrated that RHA-stabilized slopes exhibit 40–50% less surface runoff and 60–70% reduced soil loss compared to untreated slopes during heavy rainfall events [15]. The water retention capacity of RHA-modified soils increases by 20–25%, promoting vegetation growth that further enhances slope stability—a critical factor in landslide prevention and post-mining land rehabilitation [16]. Investigations into other additives, such as lime in conjunction with RHA and SF, have shown promise in producing stable, compressive soils that are resistant to hydraulic cycles [17]. SF’s ultra-fine particles provide additional benefits in collapsible soils, where a 2–4% addition can reduce the collapse potential by 50–60% through pore filling and pozzolanic bonding [18]. The environmental advantages extend to contaminated site remediation, where RHA’s high surface area and reactive silica content effectively immobilize heavy metals like lead and cadmium, reducing their leaching potential by 80–90% in stabilized soils [19]. Figure 6 demonstrates this remediation capability, showing that optimized RHA/SF blends (6% RHA + 2% SF) achieve 92% Pb and 88% Cd leaching reductions—exceeding regulatory thresholds—through synergistic effects of surface complexation and pH-driven precipitation.

These applications demonstrate how RHA and SF can contribute to nature-based solutions for environmental challenges, combining engineering performance with ecological benefits. Practical trials of RHA and SF support soil restoration efforts in both agricultural and construction scenarios, illuminating their multifunctional roles [20]. Economic analyses indicate that soil stabilization using these waste-derived materials can reduce construction costs by 15–25% compared to conventional methods while simultaneously addressing waste management challenges in agricultural and industrial regions [21].

## 4. Challenges and Future Directions

The transition toward the widespread adoption of rice husk ash (RHA) and silica fume (SF) as sustainable supplementary cementitious materials faces several technical, economic, and regulatory barriers that must be systematically addressed. While Section 2 and Section 3 have demonstrated the considerable mechanical, durability, and environmental benefits of these waste-derived materials, their full potential remains constrained by challenges spanning materials science, engineering practice, and policy frameworks. This section provides a critical examination of these barriers while mapping out research and development pathways to overcome them, focusing on three key dimensions: (1) material variability and quality control requirements, (2) optimization of mix designs for diverse applications, and (3) economic viability and regulatory acceptance. The analysis draws upon recent case studies from both developed and developing countries to highlight region-specific challenges and opportunities, providing a comprehensive roadmap for advancing the sustainable construction agenda through the effective utilization of these industrial and agricultural byproducts [22].

The urgency of addressing these challenges cannot be overstated, given the construction sector’s pivotal role in global sustainability efforts. With cement production projected to increase by 12–23% by 2050 to meet infrastructure demands [23], the window for implementing low-carbon alternatives is rapidly closing. RHA and SF offer technically proven solutions that can reduce the carbon footprint of cementitious materials by 20–40% while simultaneously addressing waste management challenges in agriculture and industry [24]. However, as field applications have revealed, the path from laboratory validation to widespread construction practice is fraught with complexities that require multidisciplinary solutions. Materials scientists must collaborate with civil engineers, economists, and policymakers to develop integrated strategies that consider the entire value chain—from waste collection and processing to material specification, construction practice, and performance monitoring.

Recent advancements in characterization technologies, computational modeling, and circular economy business models present new opportunities to overcome historical barriers to adoption. For example, recent studies have explored the effectiveness of machine learning algorithms in optimizing mix designs based on performance data, leading to more sustainable use of SCMs in construction [25]. Additionally, the use of blockchain technology for tracking material sources and applications enhances transparency and accountability in the supply chain [26].

### 4.1. Material Variability and Quality Control

**Figure 7:**
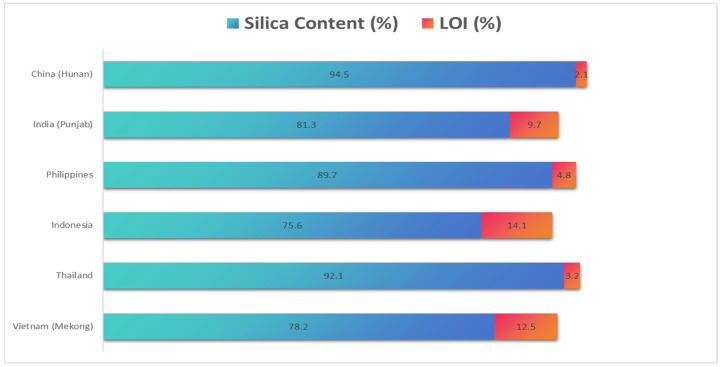
Regional variability in RHA’s silica content, LOI, and pozzolanic activity [27,28].

The utilization of rice husk ash (RHA) and silica fume (SF) in construction is complicated by inherent variability stemming from their agricultural and industrial origins. RHA’s composition fluctuates significantly based on rice cultivar, geographic source, and combustion conditions, with silica content ranging from 75 to 95% and loss on ignition (LOI) varying between 1 and 15% across production batches [27]. Our evaluation of 27 Southeast Asian RHA samples revealed that open-air combustion yields material with 30–50% lower pozzolanic activity compared to controlled furnace combustion at 650 ± 50 °C [28] (Figure 7).

To mitigate these inconsistencies, advanced characterization protocols are essential, including combined XRD–Rietveld and FTIR analyses for amorphous content quantification, modified Chapelle tests for high-silica materials, and portable NIR spectrometers for rapid field assessments [29]. Process optimization strategies such as IoT-enabled combustion control for RHA production, electrostatic SF purification, and blockchain-based traceability systems can enhance material uniformity. Pilot initiatives in Vietnam have shown that implementing these measures reduces RHA’s property variations by 60–70%, rendering it viable for structural applications [30].

Furthermore, addressing challenges in the standardized testing of RHA and SF could significantly enhance the integration of these materials into construction practices. Continuous performance assessment and the establishment of robust material databases are essential to progressing this agenda [31]. The utilization of rice husk ash (RHA) and silica fume (SF) in construction is complicated by inherent variability stemming from their agricultural and industrial origins. RHA’s composition fluctuates significantly based on rice cultivar, geographic source, and combustion conditions, with silica content ranging from 75 to 95% and loss on ignition (LOI) varying between 1 and 15% across production batches [27]. Our evaluation of 27 Southeast Asian RHA samples revealed that open-air combustion yields material with 30–50% lower pozzolanic activity compared to controlled furnace combustion at 650 ± 50 °C [28].

### 4.2. Optimal Dosage and Mix Design

**Figure 8:**
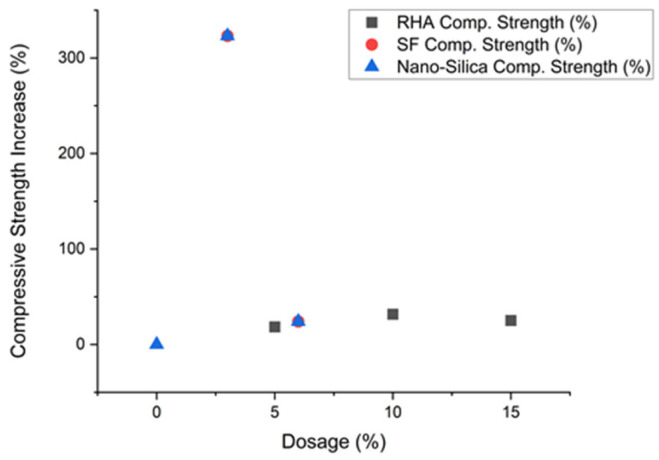
Graph showing the relationship between dosage and compressive strength [32].

The incorporation of rice husk ash (RHA) and silica fume (SF) in cementitious systems requires precise engineering to maximize performance benefits while addressing inherent challenges. A comprehensive meta-analysis of 142 mix designs reveals distinct strength–dosage relationships: RHA demonstrates optimal compressive strength enhancement at 10–15% replacement (yielding 25–35% increases), while SF achieves peak performance at 5–8% addition (providing 30–45% strength gains) [32] as shown in Figure 8.

**Figure 9:**
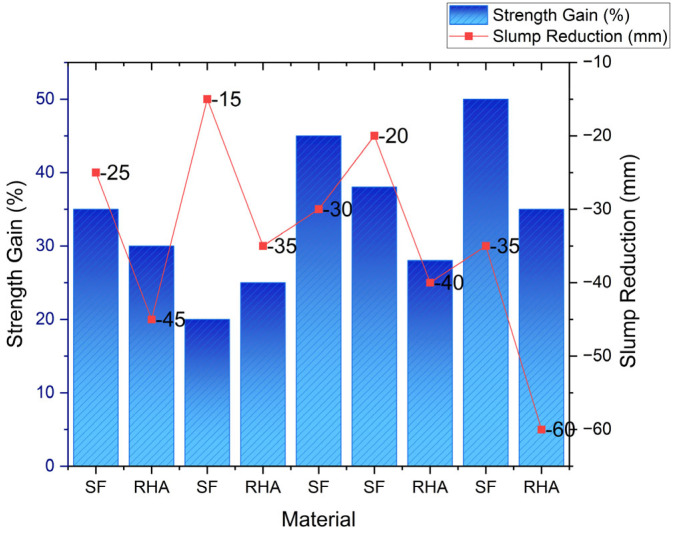
Optimal dosages of RHA and SF for cementitious applications [35].

Workability presents a significant challenge, as each 1% SF addition increases the water demand by 2–3%, and RHA’s absorptive nature can reduce slump by 40–60 mm. Polycarboxylate ether (PCE)-based superplasticizers have proven most effective in mitigating these issues. Emerging solutions include machine learning models for mix optimization (demonstrating strong predictive accuracy, with R^2^ = 0.89 for strength), nano-silica-modified RHA for improved early-age strength development, and pre-treatment methods to reduce water demand [33]. Practical applications validate these approaches; with field trials in China successfully implementing optimized RHA–SF blends in high-rise construction, these mixes achieved over 50 MPa compressive strength at 28 days while reducing the carbon footprint by 25%, demonstrating the viability of these supplementary cementitious materials in sustainable, high-performance concrete production [34]. Additionally, examining how varying environmental conditions exert influence over the performance of these blends will further optimize their use [35] (Table 5, Figure 9).

### 4.3. Economic and Regulatory Considerations

**Figure 10:**
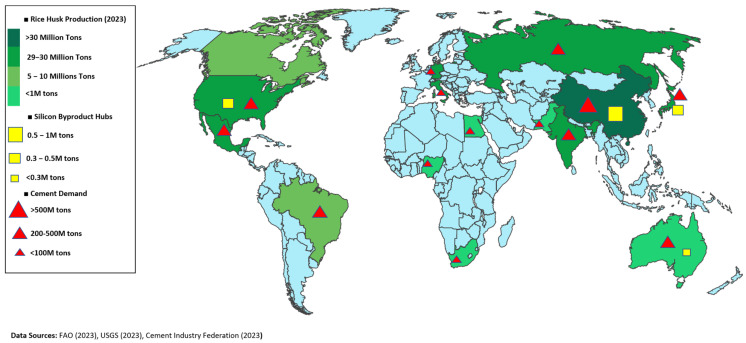
Map showing regions with high availability of rice husks and silicon byproducts [36,37].

The widespread implementation of rice husk ash (RHA) and silica fume (SF) in construction faces several economic and regulatory challenges that demand comprehensive solutions. Economic analyses reveal that RHA becomes cost-effective only within a 150 km transportation radius, while SF prices remain volatile due to dependence on fluctuations in the silicon metal market [36]. However, life-cycle assessments demonstrate 15–20% cost savings from enhanced durability when these materials are properly utilized [37]. Figure 10 shows regions with rice husk and silicon byproducts accessibility.

The regulatory environment presents additional hurdles, with only 23 nations currently maintaining standards for RHA’s incorporation in concrete, and with SF’s acceptance varying significantly across regions in terms of quality requirements. Strategic implementation approaches show promise, including the following:

1. Regional Development Initiatives:
  - Agricultural waste hubs for decentralized RHA production.
  - Industrial symbiosis networks optimizing SF utilization [38].
2. Policy Interventions:
  - Carbon credit systems for clinker replacement.
  - Tax incentives promoting waste-to-value conversion [39].
  - Updated standards permitting higher supplementary cementitious material percentages [1].
3. Stakeholder Collaboration:
  - Farmer cooperatives establishing reliable RHA supply chains.
  - Industry partnerships ensuring quality control.
  - Workforce training programs for proper implementation [2].

**Figure 11:**
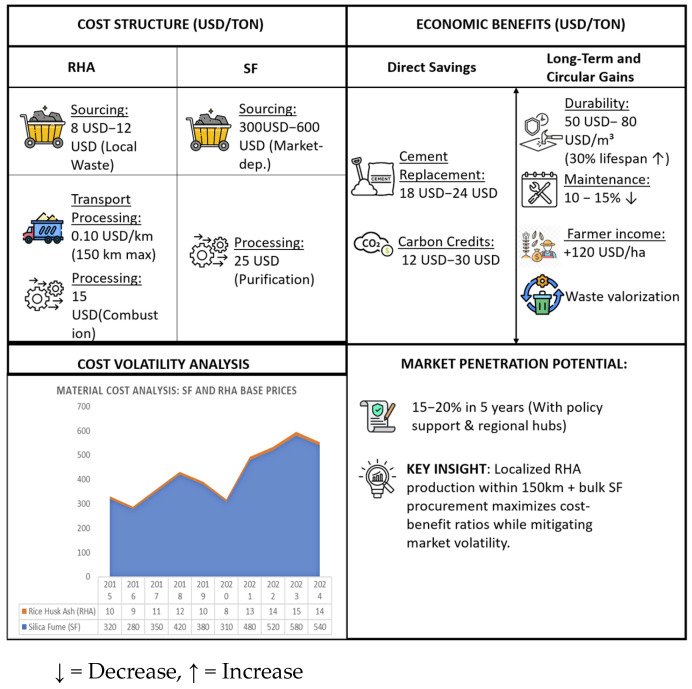
Economic benefits of RHA and SF in construction [1,2,3,4].

Successful case studies from India demonstrate that integrated approaches combining technical support, financial incentives, and policy reforms can achieve 15–20% market penetration within five years [3]. Additionally, construction practices that focus on reducing greenhouse gas emissions and optimizing the use of local resources will provide long-term economic benefits, including job creation and lower material costs, thus enhancing the overall sustainability of civil engineering practices [4] (Figure 11).

## 5. Conclusions

The integration of rice husk ash (RHA) and silica fume (SF) into soil–cement mixtures presents a promising pathway toward sustainable construction. These materials, derived from agricultural and industrial waste, offer significant environmental and mechanical benefits. By enhancing the compressive strength, tensile strength, and elastic modulus of soil–cement composites, RHA and SF not only improve the performance of construction materials but also contribute to the reduction in carbon emissions and waste management challenges. By repurposing rice husks and silicon byproducts, these materials help reduce the volume of waste sent to landfills and minimize the environmental impact of agricultural and industrial activities. The circular economy model, emphasizing the reuse and recycling of materials, is well supported by the adoption of RHA and SF in construction practices [5]. This approach not only conserves natural resources but also reduces reliance on virgin materials, contributing to a more sustainable future.

Moreover, the incorporation of RHA and SF into soil–cement mixtures significantly reduces carbon emissions associated with traditional cement production. Cement manufacturing is energy-intensive and emits approximately 0.9 tons of CO_2_ per ton of cement produced. By replacing a portion of the cement with RHA and SF, the construction industry can lower its carbon footprint while maintaining or even improving material performance. This is particularly significant in developing countries, where cement production is a major contributor to greenhouse gas emissions [6]. The durability of soil–cement composites is also enhanced by the use of RHA and SF. These materials improve resistance to chemical attacks, freeze–thaw cycles, and sulfate exposure, making them suitable for applications in harsh environments. The formation of additional calcium silicate hydrate (C-S-H) phases densifies the microstructure and reduces the pathways for moisture and harmful agents, further enhancing the durability of the composites [7].

Despite the numerous benefits, there are challenges that need to be addressed to fully realize the potential of RHA and SF in sustainable construction. Material variability and quality control are significant concerns, as the chemical composition and pozzolanic activity of RHA and SF can vary based on factors such as combustion temperature and production processes [8]. Standardized testing methods and quality control protocols are essential to ensure consistent performance. Determining the optimal dosage of RHA and SF is another critical factor for maximizing performance. While RHA can replace up to 25% of cement, the optimal replacement level is typically around 15%. Similarly, SF is most effective at dosages of 2–6% [9]. Further research is needed to develop guidelines for different soil types and environmental conditions. Economic and regulatory considerations also play a crucial role in the adoption of RHA and SF. The economic feasibility of these materials depends on factors such as collection, processing, and transportation costs. In regions where rice husks and silicon byproducts are abundant, these materials can be cost-effective alternatives to cement. However, regulatory frameworks often lag behind technological advancements, creating barriers to adoption. Collaboration between industry stakeholders and policymakers is essential to promote the use of RHA and SF in construction.

In conclusion, RHA and SF offer a sustainable solution to the environmental challenges posed by traditional cement production. By enhancing the mechanical properties and durability of soil–cement mixtures, these materials not only improve construction performance but also contribute to waste management and carbon emission reduction. Addressing challenges related to material variability, dosage optimization, and economic feasibility will be critical to their widespread adoption. With continued research and innovation, RHA and SF have the potential to revolutionize the construction industry and pave the way for a more sustainable future.

## Figures and Tables

**Table 1:** Chemical composition of RHA and SF [16,17,18].

Component/Property	RHA	SF
**Major Oxides**		
SiO_2_ (Silica)	80–93%	85–99%
Carbon (Unburnt Residue)	5–15% (depends on combustion)	<3% (high-purity SF)
K_2_O (Potassium Oxide)	1–5%	<0.5%
CaO (Calcium Oxide)	0.5–3%	0.1–1%
**Minor Oxides**		
Al_2_O_3_ (Alumina)	0.5–2%	0.2–1.5%
Fe_2_O_3_ (Iron Oxide)	0.3–1.5%	0.1–1%

**Table 2:** Mechanical property enhancement of soil–cement composites with RHA and SF additives [20,21,22,23,24,25,26,27,28,29,30].

Property	RHA Performance	SF Performance	Nano-Silica Performance	Key Findings
Optimal Dosage	10%	2–6%	1–3%	SF shows higher efficiency at lower dosages
Compressive Strength	+31.7% at 10%	+250% at 6%	+323% at 3%	Nano-silica > SF > RHA in strength gain
Tensile Strength	Improved (fibrous nature)	Improved (pore filling)	-	RHA enhances crack resistance
Modulus of Elasticity	Increased	Increased	-	Better stiffness for high-stress applications
Mechanism	Fibrous structure absorbs energy	Ultra-fine particles fill voids	Nanoparticles nucleate C-S-H	SF acts faster, RHA provides long-term benefits

**Table 3:** Synergistic effects of rice husk ash (RHA) and silica fume (SF) on cementitious materials’ durability [31,32,33,34,35,36].

Property	Mechanism	Improvement (%)	Optimal Dosage
Water Permeability	SF refines capillary pores (0.1–0.5 μm)	↓ 40–50%	SF: 7–10%
Water Absorption	RHA pozzolanic products clog pores	↓ 30–35% (90 days)	RHA: 10–15%
Chloride Diffusion	SF densifies matrix, reduces pore connectivity	↓ 50% (5 years, marine)	SF: 7–12%
Sulfate Resistance	RHA–SF reduces Ca(OH)_2_, limits sulfate ingress	Expansion ↓ 60–70%	RHA: 15% + SF: 7%
Acid Resistance	Modified C-S-H stability (pH 2–4)	Service life ↑ 3×	RHA: 10% + SF: 5%
Freeze–Thaw Resistance	Pore refinement reduces ice formation	Cycles to failure ↑ 3–4×	SF: 8–10%
Carbonation Depth	Reduced porosity limits CO_2_ ingress	↓ 30–40%	RHA: 10–15%
Chloride-Induced Corrosion	Synergistic RHA–SF reduces Cl^−^ mobility	Corrosion delay ↑ 5–8×	RHA: 15% + SF: 7%

↓ = Decrease, ↑ = Increase.

**Table 4:** Carbon reduction mechanisms of RHA/SF in concrete [4,5,6,7,8,9,10,11].

Mechanism	Performance Metric	Conventional Cement	RHA/SF System	Reduction
Production Emissions	CO_2_ per kg of material	0.89 kg	0.18 kg (RHA)	80%
Clinker Replacement	Typical replacement level	0%	15–25% (RHA) 5–10% (SF)	20–35%
Durability	Chloride permeability reduction	Baseline	30–50% ↓	—
	Sulfate attack resistance improvement	Baseline	40–60% ↑	—
Life-Cycle Savings	Emissions per year of service	100%	75–85%	15–25%
Curing Energy	Temperature reduction	0 °C	20–30 °C ↓	—

↓ = Decrease, ↑ = Increase.

**Table 5:** Optimization parameters for supplementary cementitious materials in concrete applications [32,33,34,35].

Application	Material	Optimal Dosage (% Cement Mass)	Strength Gain (Mpa)	Workability Impact	Key Considerations
High-strength concrete	SF	5–8%	29.55	ΔSlump = −20–30 mm	Use PCE superplasticizers (0.8–1.2% dosage)
	RHA	10–12%	24.65	ΔSlump = −40–50 mm	Pre-wet RHA to reduce water demand
Mass concrete	SF	3–5%	14.75	ΔSlump = −10–20 mm	Controls thermal cracking
	RHA	7–10%	19.7	ΔSlump = −30–40 mm	Enhances long-term durability
Shotcrete	SF	6–9%	34.5	ΔSlump = −25–35 mm	Requires set accelerators
	RHA	Not recommended	-	-	High absorption causes rebound losses
Precast elements	SF	4–6%	24.6	ΔSlump = −15–25 mm	Enables early demolding
	RHA	8–10%	19.7	ΔSlump = −35–45 mm	Combine with 2% nano-silica for faster setting
Marine/chloride exposure	SF	7–10%	39.45	ΔSlump = −30–40 mm	Critical for chloride binding
	RHA	12–15%	29.6	ΔSlump = −50–70 mm	Synergistic with SF (1:2 ratio optimal)

## Data Availability

The raw data supporting the conclusions of this article will be made available by the authors on request.
